# High-throughput simultaneous screen and counterscreen identifies homoharringtonine as synthetic lethal with von Hippel-Lindau loss in renal cell carcinoma

**DOI:** 10.18632/oncotarget.4773

**Published:** 2015-07-03

**Authors:** Nicholas C. Wolff, Andrea Pavía-Jiménez, Vanina T. Tcheuyap, Shane Alexander, Mridula Vishwanath, Alana Christie, Xian-Jin Xie, Noelle S. Williams, Payal Kapur, Bruce Posner, Renée M. McKay, James Brugarolas

**Affiliations:** ^1^ Kidney Cancer Program, Simmons Comprehensive Cancer Center, University of Texas Southwestern Medical Center, Dallas, Texas, USA; ^2^ Department of Internal Medicine - Hematology-Oncology Division, University of Texas Southwestern Medical Center, Dallas, Texas, USA; ^3^ Department of Developmental Biology, University of Texas Southwestern Medical Center, Dallas, Texas, USA; ^4^ Department of Biochemistry, University of Texas Southwestern Medical Center, Dallas, Texas, USA; ^5^ Department of Pathology, University of Texas Southwestern Medical Center, Dallas, Texas, USA; ^6^ BioTek Instruments, Winooski, Vermont, USA

**Keywords:** high-content drug screen, omacetaxine mepesuccinate, patiend-derived xenografts, tumorgrafts

## Abstract

Renal cell carcinoma (RCC) accounts for 85% of primary renal neoplasms, and is rarely curable when metastatic. Approximately 70% of RCCs are clear-cell type (ccRCC), and in >80% the von Hippel-Lindau (*VHL*) gene is mutated or silenced. We developed a novel, high-content, screening strategy for the identification of small molecules that are synthetic lethal with genes mutated in cancer. In this strategy, the screen and counterscreen are conducted simultaneously by differentially labeling mutant and reconstituted isogenic tumor cell line pairs with different fluorochromes and using a highly sensitive high-throughput imaging-based platform. This approach minimizes confounding factors from sequential screening, and more accurately replicates the *in vivo* cancer setting where cancer cells are adjacent to normal cells. A screen of ~12,800 small molecules identified homoharringtonine (HHT), an FDA-approved drug for treating chronic myeloid leukemia, as a *VHL*-synthetic lethal agent in ccRCC. HHT induced apoptosis in *VHL-*mutant, but not *VHL-*reconstituted, ccRCC cells, and inhibited tumor growth in 30% of *VHL*-mutant patient-derived ccRCC tumorgraft lines tested. Building on a novel screening strategy and utilizing a validated RCC tumorgraft model recapitulating the genetics and drug responsiveness of human RCC, these studies identify HHT as a potential therapeutic agent for a subset of *VHL*-deficient ccRCCs.

## INTRODUCTION

Renal cell carcinoma is the most common form of kidney cancer, with roughly 60,000 new cases reported each year [1], and clear cell renal cell carcinoma (ccRCC) represents ~70% of all RCCs. The mainstay treatment for localized ccRCC is surgery, and five-year survival rates range from 62-97% depending on the size of the tumor [2]. However, once the cancer has metastasized to other organs, the five-year survival rate is much lower, at only 8-41% [2]. As ccRCC is resistant to standard cancer treatments such as chemotherapy and radiation, treatment options, until recently, were greatly limited [3].

The von Hippel-Lindau (*VHL*) gene, which encodes an E3 ubiquitin ligase, is inactivated in > 80% of all ccRCCs [4, 5]. One consequence of *VHL* inactivation is the upregulation of hypoxia-inducible factor-2 alpha (HIF-2α) and subsequently, the upregulation of vascular endothelial growth factor (VEGF), which leads to increased angiogenesis, a critical component of tumorigenesis [6]. Additionally, mammalian target of rapamycin complex 1 (mTORC1), a serine/threonine kinase that is a key regulator of protein translation and cell proliferation, is also activated in RCC [7, 8]. Recent FDA-approved drugs for the treatment of ccRCC include drugs that target effector pathways downstream of *VHL* such as the VEGF pathway (bevacizumab, sorafenib, sunitinib, pazopanib, axitinib) and the mTORC1 pathway (temsirolimus and everolimus) [9]. Both temsirolimus and everolimus are analogues of sirolimus (also called rapamycin), and in patients temsirolimus is metabolized to sirolimus, which accounts for 70% of the circulating drug [10, 11]. While sequential use of these drugs has improved outcomes, disease progression is typically only delayed by a few months and most patients eventually develop resistance to these drugs [12, 13]. The identification of novel drugs (and/or drugs that synergize with existing drugs) to treat ccRCC patients is therefore critical for improved patient outcomes.

The concept of “synthetic lethality” is being successfully exploited in the cancer field to identify drugs that specifically target cancer cells while leaving healthy, non-cancer cells unharmed [14-16]. Synthetic lethal drugs often target functions that are essential for survival in the presence of a gene mutated in cancer cells [15, 17]. Synthetic lethal chemical or RNAi screens performed on cancer cell lines with a known mutation have been successful in identifying such agents. For example, synthetic lethal screens have identified Poly(ADP-ribose) polymerase (PARP) inhibitors as being potentially effective in treating breast and ovarian cancers that harbor BRCA1 or BRCA2 mutations [18], and compounds that are synthetic lethal with RAS mutations found in colon, lung, and other cancers have also been identified via such screens [19-21].

To identify small molecule drugs that exhibit synthetic lethality with the *VHL* gene and thus could potentially be widely applicable for the treatment of RCC, we performed a high-throughput chemical screen. The design of this cell-based screen included the following important features: 1) It employed a *VHL*-mutant RCC tumor cell line and an isogenic control reconstituted with *VHL*. 2) The screen (involving mutant cells) and the counterscreen (with the reconstituted cells) were carried out simultaneously. This multiplexed screening strategy, which was enabled by the differential labeling of the two cell populations with distinctive fluorochromes, minimizes confounding variables that can arise from sequential screening, and more accurately replicates the *in vivo* cancer setting where cancer cells are adjacent to normal cells. 3) It utilized an image-based screening platform that provides high-content information of drug effects on the two cell populations. 4) Our versatile platform is amenable not only to the identification of synthetic lethal compounds, but also compounds that are synergistic with existing drugs. 5) Follow-up studies take advantage of a state-of-the-art RCC tumorgraft platform that evaluates the activity of candidate drugs against patient tumor samples implanted into immunocompromised mice that reproduce the responsiveness of ccRCC to drugs in the clinic [22].

Here, we report the identification of homoharringtonine (HHT) as a synthetic lethal compound effective against *VHL*-deficient ccRCC cells in our screen. HHT is a plant alkaloid that has been studied for many decades as an anti-leukemic agent (particularly in China), and in 2012, a semi-synthetic form of HHT (ssHHT; omacetaxine mepesuccinate) was approved by the FDA for the treatment of chronic myeloid leukemia (CML) [23]. Testing of HHT in our validated tumorgraft model showed that it was able to inhibit tumor growth in 30% of the tumorgraft lines when tested at nanomolar, clinically-relevant concentrations [24, 25]. The identification and validation in tumorgrafts of HHT provides a proof-of-principle for this screening strategy.

## RESULTS

### Novel platform to identify small molecule compounds synthetic lethal with *VHL* mutation

**Figure 1:**
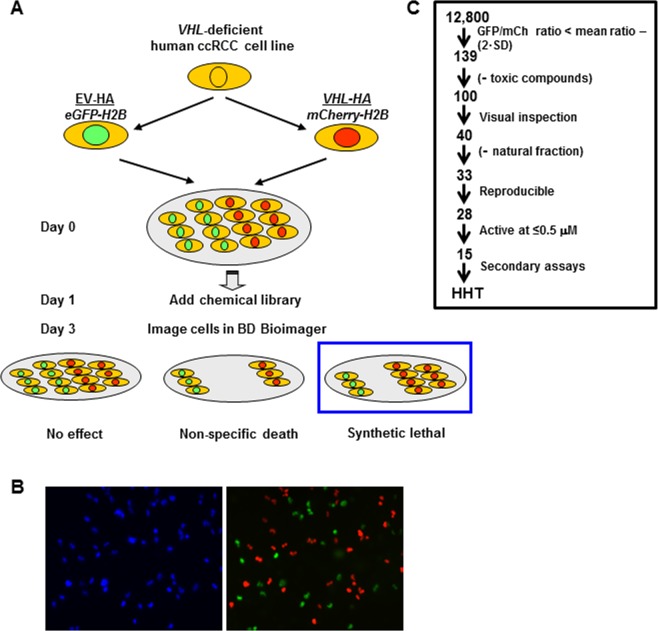
Screening strategy to identify compounds that are synthetic lethal with *VHL* **A**. Schematic diagram of a ~12,800 chemical library synthetic lethal screen. *VHL*-deficient 786-O cells stably transduced with an empty vector control (EV) or a *VHL*-expressing retrovirus (*VHL*-HA) were differentially labeled with eGFP-Histone H2B (eGFP-H2B) and mCherry-tagged H2B respectively, and after 24 hours the chemical library was dispensed (final concentration of 5 μM) and cells cultured for 48 hours in the presence of 2 nM rapamycin. Fluorescence imaging (using the BD Pathway 855 imager) was used to identify compounds preferentially depleting *VHL*-deficient/GFP-positive cells after cells were segmented. **B**. Left panel: Representative image of fluorescence signal from Hoescht-labeled 786-O cells. Right panel: overlay of GFP fluorescence signal (green; from 786-O EV^GFP^ cells) and mCherry fluorescence nuclear signal (red; *VHL*^mCh^ cells). **C**. Flow chart showing the selection criteria utilized to narrow down compounds for further analysis. Toxicity was determined as ≤100 *VHL*^mCh^ cells per field. SD, standard deviation.

To identify small molecule compounds synthetic lethal with *VHL*, we carried out an unbiased cell-based screen of a chemical library of ~12,800 compounds using 786-O cells, an extensively studied cell line derived from a *VHL*-deficient primary human ccRCC [26]. 786-O cells were stably transfected with either an empty vector (EV) control or reconstituted with an HA-tagged *VHL* cDNA. *VHL* reconstitution downregulated HIF-2α and GLUT1 as expected (Supplementary Figure 1A). To distinguish between the two otherwise isogenic cell lines, we transduced the *VHL*-deficient 786-O cells with a histone H2B-GFP reporter and the *VHL*-reconstituted cells with an H2B-mCherry reporter. The histone proteins drive the fluorochrome to nuclei facilitating cell identification and segmentation algorithms (Figure 1A and 1B). EV control (EV^GFP^) and *VHL*-reconstituted cells (*VHL*^mCh^) were mixed together 1:1 and dispensed into wells (Figure 1A and 1B).

It has been previously reported that 786-O cells reconstituted with *VHL* grow at a similar rate in culture as *VHL*-deficient parental cells (though there is a clear growth difference in xenografts *in vivo*) [26]. As a similar proliferation rate for both cell lines was critical to the design and accuracy of our screen, we confirmed that 786-O *VHL*^mCh^ cells grew at a similar rate as 786-O EV^GFP^ cells. After 3 days in culture (which corresponded to the duration of the chemical compound screen), we observed similar numbers of 786-O *VHL*^mCh^ and 786-O EV^GFP^ cells (Supplementary Figure 1B).

A key advantage of this dual screen design is that the screen and the counterscreen are conducted simultaneously using a fluorescence-based readout, thereby maximizing sensitivity and minimizing confounding variables that can occur when screens are carried out at different times and in different tissue culture plates. To determine the sensitivity of our screening platform, we evaluated defined ratios of *VHL*^mCh^ and EV^GFP^ cells using a BD Pathway 855 imager. Small changes in cell population ratios (i.e. 90:10, 80:20, etc.) were readily and accurately detected with the BD Pathway imaging system (Supplementary Figure 1C). However, statistical analysis of the signal readout versus the number of cells plated revealed a skewing toward the GFP signal, such that any given percent of green value read is likely to be 4.51±4.60 percentage points greater than was plated. As this was found to be stable for each ratio, and skewed in favor of the EV^GFP^ cells, this should not compromise the results of the screen, and in fact would require a more stringent decrease of the EV^GFP^ cells upon drug treatment to indicate cell killing.

### Homoharringtonine (HHT) acts as a synthetic lethal compound to preferentially kill *VHL*-deficient 786-O cells

We screened ~12,800 compounds including the Prestwick Chemical Library^®^ of all FDA-approved drugs, as well as an NIH Collection of experimental drugs (Supplementary Figure 2A) at 5 μM in 40 384-well plates with appropriate controls (Supplementary Figure 2A and 2B). The average Z’ score for the plates was 0.674 (+/− 0.072 standard deviation), which is an excellent signal-to-noise value. A low concentration of sirolimus (2 nM) was included in the screen, thus potentially allowing for the identification of molecules that are synergistic with sirolimus (which may lead to more effective drug combinations). We chose sirolimus because it is a first line treatment for ccRCC, and also because it acts directly on the cancer cell as opposed to anti-angiogenic drugs which act on endothelial cells. We identified 139 compounds that differentially depleted GFP-positive (*VHL*-deficient) cells with ratios of EV^GFP^*/VHL*^mCh^ lower than 2 times the standard deviation of the mean ratio for the library. Compounds that were highly toxic ( < 100 *VHL*^mCh^ cells per well) were eliminated (*n* = 39) (Figure 1C). After visual inspection of the images from hit wells, 40 compounds were selected for further analyses. They included 7 mixtures of natural compounds (natural fractions), and among the remaining 33 compounds, 28 were reproducible and 15 had activity at concentrations below 0.5 μM (Figure 1C). None of the compounds were synergistic with sirolimus, and further analyses focused on homoharringtonine (HHT), an NIH-approved drug [23] that could be repurposed for ccRCC (Figure 1C).

**Figure 2:**
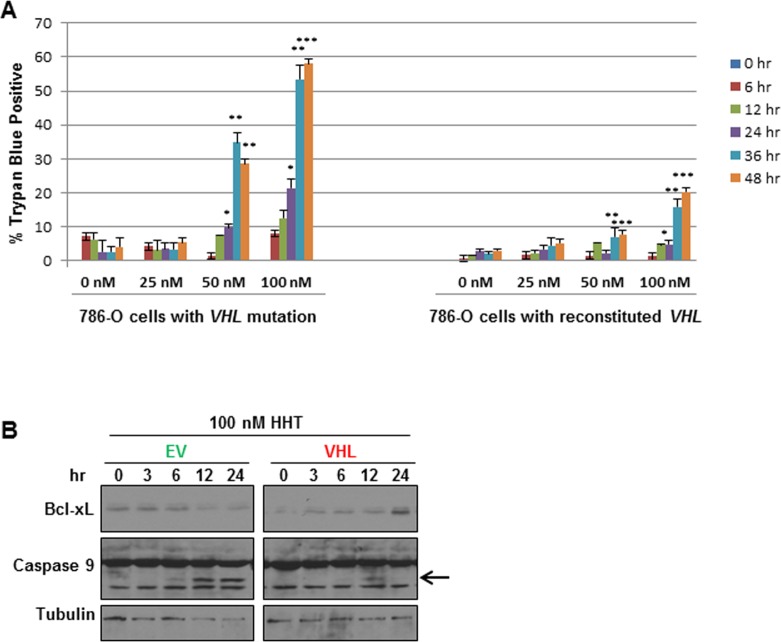
Homoharringtonine (HHT) preferentially induces cell death in *VHL*-deficient 786-O cells **A**. 786-O cells with *VHL* mutation (green) or with *VHL*-reconstituted (red) were treated with different concentrations of HHT (0, 25, 50, or 100 nM) for 0, 6, 12, 24, 36, or 48 hours, and cell death was measured by trypan blue exclusion assay (*n* = 3 for each concentration and time point; *p < 0.05, **p < 0.001, ***p < 0.0001 between 786-O EV cells and 786-O with VHL reconstituted at the same concentration and time point). **B**. Western blot analysis of 786-O EV^GFP^ (EV) or *VHL*^mCh^ (VHL) cells treated with 100 nM HHT for 0, 3, 6, 12, or 24 hours. Blots were probed for Bcl-xL and Caspase 9, and α-Tubulin as loading control. Arrow indicates the cleaved Caspase 9 product.

At low nanomolar concentrations, HHT preferentially killed *VHL*-deficient cells in a dose-dependent manner (Figure 2A). At 50 nM, 30-40% of the *VHL*-deficient cells were killed by 36 hours. At the same time point, fewer than 10% of *VHL*-reconstituted cells were dead. At 100 nM, the number of dead *VHL*-deficient cells reached 50-60%, but was less than 25% for the *VHL*-reconstituted cells (Figure 2A). Importantly, 50 nM is a concentration achievable in patients [24]. This effect was independent of sirolimus (data not shown). Interestingly, HHT induced apoptosis preferentially in *VHL*-deficient cells: caspase 9 cleavage was observed at 50 nM and 100 nM HHT treatment within 12 hours in *VHL*-deficient cells, but not in *VHL*-reconstituted cells (Figure 2B and data not shown). Additionally, we found that Bcl-xL, a member of the anti-apoptotic Bcl-2 family whose expression has been shown to be dependent upon VHL expression in the context of chemically-induced hypoxia in 786-O cells [27], was upregulated at 24 hours in HHT-treated *VHL*-reconstituted but not *VHL*-deficient cells. Together these data indicate that 50 nM HHT can effectively kill *VHL*-deficient 786-O cells, most likely by inducing apoptosis.

### HHT is active against *VHL*-deficient RCC in tumorgraft model

To determine whether HHT may have activity against RCC, we evaluated its activity in a tumorgraft model of RCC that we previously showed recapitulates the histology, gene expression, and drug responsiveness of human RCC [22]. Pharmacokinetic (PK) analyses were first performed to determine the dosage required to achieve plasma concentrations of ~50 nM HHT, which are reached in the treatment of CML in patients [24, 25].

We evaluated PK parameters after administration of a single dose of HHT by oral gavage to mice of 0.4 mg/kg, 0.7 mg/kg and 2 mg/kg (Table 1). C_max_ was reached at 10 minutes (first time point sampled) for the two lower doses but increased to 1.5 hours at the higher 2 mg/kg dose. Terminal t½ also increased with increasing dose. Treatment with 0.4 mg/kg HTT by gavage most closely approximated human exposures [25]. However, the half-life in humans, where it is dosed subcutaneously, was 7 h, whereas it was 1.86 h in mice (at 0.4 mg/kg). The half-life increased to 2.8 h at the next dose level (0.7 mg/kg). While this resulted in exposures that were higher than in humans, HHT concentrations above 50 nM (equivalent to 27.3 ng/mL; MW=545.6) were sustained for ~6 hours. Given our in vitro studies, we sought to sustain 50 nM drug levels and mice were dosed with 0.7 mg/kg HHT given by gavage twice daily.

**Table 1:** Pharmacokinetic analysis of HHT in mice

	0.4 mg/kg	0.7 mg/kg	2 mg/kg
Terminal t^½^ (hr)	1.86	2.80	6.74
T_max_ (hr)	0.17	0.17	1.50
C_max_ (ng/ml)	28.8	57.8	97.2
AUC_inf_ (hr*ng/ml)	251.0	439.8	905.2
Vz/F (ml)	87.7	123.2	453.3
Cl/F (ml/hr)	32.7	30.5	46.6

Terminal t½: terminal half-life; T_max_: time to maximal drug concentration; C_max_: maximal drug concentration; AUC_inf_: area under the concentration time curve (AUC) from time 0 to infinity; Vz/F: apparent volume of distribution; Cl/F: apparent total body clearance. PK parameters were determined using the noncompartmental analysis tool in Phoenix WinNonlin.

**Figure 3:**
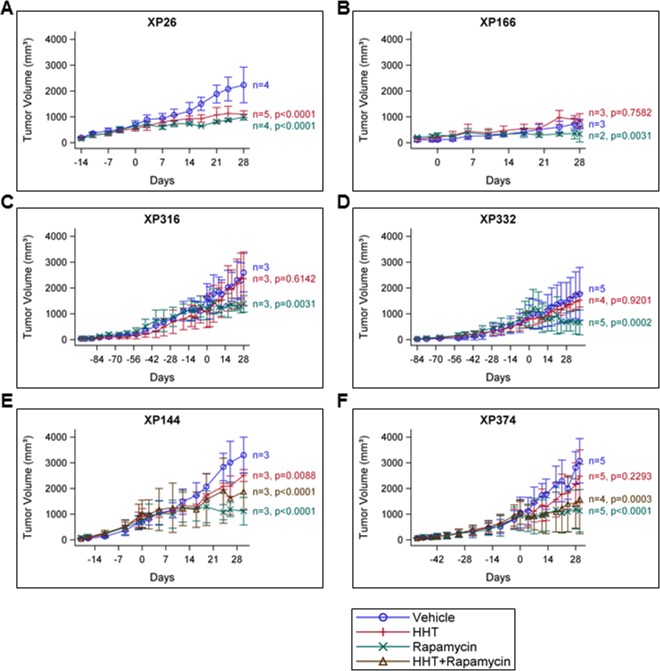
HHT inhibits tumor growth in a ccRCC tumorgraft model **A-F**, Tumorgraft lines were treated with vehicle control, HHT (0.7 mg/kg), rapamycin (0.5 mg/kg), (or the combination, where indicated) and tumor growth was measured on the indicated days. Day 0 represents the first day of treatment.

We selected 6 independently-derived ccRCC tumorgraft lines that were confirmed to have *VHL* mutation. The mice (3-5 mice per tumorgraft line) were treated with either HHT (0.7 mg/kg), vehicle (as a negative control), or rapamycin (0.5 mg/kg; as a positive control). In total, 65 tumorgraft bearing mice were evaluated for these experiments in time courses lasting ~28 days, with tumor growth measured every 3 or 4 days. Most lines responded to rapamycin treatment, as expected (Figure 3A-3F).

**Figure 4:**
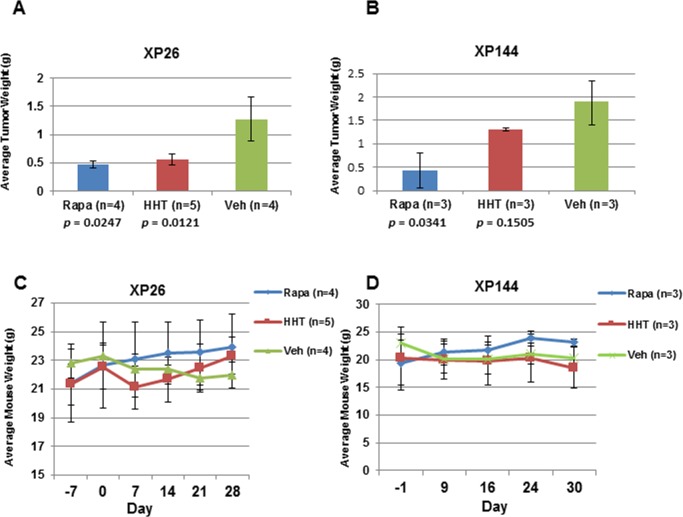
HHT treatment inhibits tumor growth with no severe toxicity **A.** and **B**. Average weight of tumors in grams (g) harvested from XP26 and XP144 tumorgraft lines treated with vehicle (Veh), homoharringtonine (HHT; 0/7 mg/kg), or rapamycin (Rapa; 0.5 mg/kg). **C.** and **D**. Average weight of XP26 and XP144 cohort mice in grams (g) taken at the indicated days prior to and over the course of the indicated drug treatment. Day 0 represents the first day of treatment.

**Figure 5:**
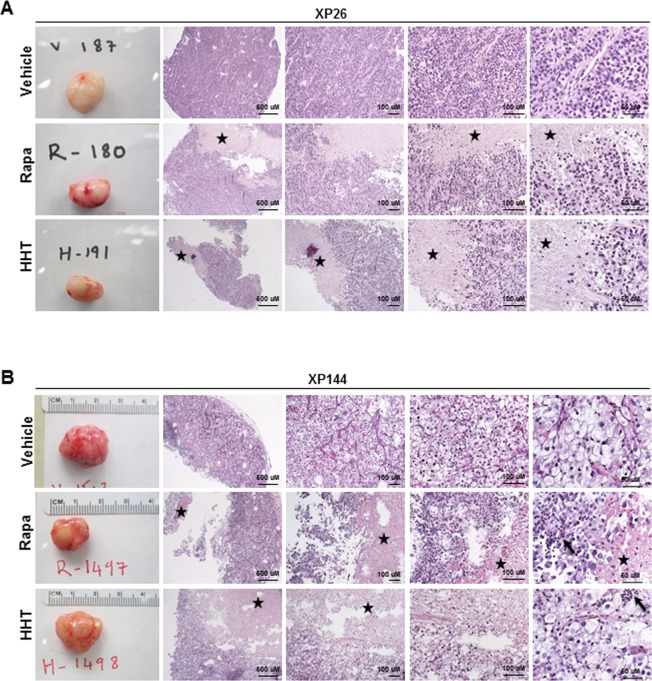
Analysis of tumors from HHT-responsive tumorgraft lines, XP26 and XP144 **A**. and **B**. Macroscopic (first panel in each row) and microscopic (next four panels of each row: progressively higher magnification) hematoxylin and eosin stained images of representative tumors harvested from tumorgraft lines XP26 (A) and XP144 (B) treated with vehicle, rapamycin (Rapa), or homoharringtonine (HHT). Tumor necrosis (depicted by star) and associated neutrophilic infiltrate (depicted by arrow) is more pronounced in tumors treated with HHT and is observed in those treated with Rapa but not seen in tumors treated with vehicle.

Of the six lines tested, two tumorgraft lines, XP26 and XP144, showed observable inhibition of tumor growth upon treatment with HHT (Figure 3A and 3E). For XP26 tumors, tumor growth (as measured by tumor volume) was inhibited by 63.7% in HHT-treated mice in comparison to vehicle treated mice, while in XP144 mice tumor growth was inhibited by 43.0%. Tumors weights from HHT-treated XP26 and XP144 mice were 56% and 32% smaller, respectively, than those from vehicle-treated mice (Figure 4A and 4B). As expected, we did not observe synergistic effects between HHT and rapamycin in the tumorgrafts. Although the HHT-treated mice maintained similar body weight compared to controls (Figure 4C and 4D), they did exhibit some signs of toxicity including rough coats and lethargy (not shown). Histological analysis of the tumors harvested from XP26 and XP144 cohorts revealed that HHT treatment induced tumor cell necrosis (Figure 5A and 5B). Hematoxylin and eosin stained sections of these tumors showed irregular geographic areas of coagulative tumor necrosis with peripherally preserved viable tumor cells and abundant karyorrhexis debris. In addition, in XP144 a neutrophilic infiltrate was observed. Intratumoral interstitial fibrosis was observed in areas with less prominent necrosis (not shown). Similar findings were observed in the rapamycin-treated tumors. In contrast, vehicle-treated tumors showed compact nests of ccRCC cells without necrosis.

Together, these data demonstrate that HHT can inhibit tumor growth in a tumorgraft RCC model using clinically-relevant regimens.

## DISCUSSION

Herein, we report the identification, using a novel screening platform, of a VHL synthetic lethal compound active in a ccRCC cell line that we subsequently validate in a state-of-the-art preclinical model. This establishes a proof-of-principle for the screening strategy we developed. The screen has several important features. First, the synthetic lethal target, *VHL*, is inactivated in over 80% of ccRCC, and therefore any compounds identified in our screen have the potential to be broadly active against ccRCC. Second, the screen and counterscreen are carried out simultaneously, which limits confounding variables from sequential screens. In addition, differential cell labeling using fluorescently-tagged nuclear (as opposed to cytosolic) proteins is optimal for cell segmentation and offers excellent accuracy. Third, this platform has the potential to identify not only synthetic lethal compounds, but also molecules that are synergistic with other drugs, such as rapamycin, which may lead to novel and more efficacious drug combinations. (Although these types of molecules may not be very abundant and we did not identify any in our library). Fourth, the library includes FDA-approved drugs as well as drugs in clinical trials, which have been extensively characterized and could be repurposed for other applications. Finally, coupling of the screen with a validated tumorgraft platform and PK analyses that ensure testing at clinically-relevant concentrations maximizes opportunities for the identification of clinically-relevant compounds.

The identification of HHT in our screen demonstrates that we can successfully find drugs that act as synthetic lethal agents when combined with *VHL* mutation using this strategy. HHT is a natural plant alkaloid derived from the coniferous tree *Cephalotaxus harringtonia* that has been reported to kill tumor cells by inducing apoptosis [28-31]. We chose to follow-up on this compound for the following reasons: 1) It offered the opportunity to evaluate a drug that has been extensively characterized and could be repurposed for renal cancer. HHT has been evaluated primarily in CML, where it was shown to lead to both major and complete cytogenetic responses [23, 32]. Following FDA approval of imatinib in 2001, efforts to develop HHT for treatment of CML slackened [33], but the development of a semisynthetic form of HHT and the finding that it could be effective in imatinib-resistant CML renewed clinical interest, eventually leading to its FDA approval for CML [33]. 2) HHT killed *VHL*-deficient cells in our tumorgraft models at concentrations achievable in patients. 3) Only a small number of RCC patients have been evaluated for HHT treatment: the only reported trial, conducted in 1996, was a small phase II clinical trial in 14 patients with advanced ccRCC, and was halted due to toxicity [34]. Thus, HHT efficacy against ccRCC has not been adequately studied. 4) We were intrigued by the observation that HHT inhibits protein synthesis [30, 35], and by the possibility that VHL loss may synergize with protein synthesis inhibitors [36]. 5) HHT penetrates the blood-brain barrier [37], and may be active against brain metastases, which are a challenge in renal cancer patients.

We found that HHT induced apoptosis in *VHL*-deficient ccRCC cells in culture but not in cells reconstituted with *VHL* at 50-100 nM concentrations. In the context of CML, studies have shown that HHT/ssHHT most likely act by inhibiting protein synthesis, and also by increasing apoptosis, in part by increasing turnover of myeloid cell leukemia 1 (MCL-1), an anti-apoptotic protein of the Bcl-2 family [29, 30]. Consistent with this, we observed an increase in Caspase 9 expression in HHT-treated 786-O cells, as well as morphologic tumor necrosis. Additionally, the observation that the anti-apoptotic protein Bcl-xL is not upregulated in our *VHL*-deficient cells may indicate a potential mechanism by which HHT is synergizing with *VHL* mutation to kill the *VHL*-deficient 786-O cells.

Subsequent studies in our tumorgraft model showed that HHT effectively inhibits tumor growth in 2/6 tumorgraft lines tested, where it induced extensive coagulative necrosis. The most likely explanation for the lack of response in all the tumorgrafts is that the effect of HHT is dependent on other factors besides *VHL*. While our studies show that HHT preferentially kills *VHL*-deficient 786-O cells, we speculate that other factors in both this cell line and the sensitive tumorgrafts determined its responsiveness to HHT. Thus, while VHL loss appears to be necessary (at least in ccRCC), it is not sufficient for HHT activity. The existence of other factors that determine HHT activity is clear based on its effectiveness against CML, which lacks mutations in *VHL*. However, our preclinical evaluation and validation of the synthetic lethality of HHT with *VHL* mutation in two tumorgraft lines represents proof-of-principle that this screening strategy can successfully identify compounds that may be useful clinically.

HHT represents the latest addition in synthetic lethal screens for ccRCC. Previously, two other screens designed to identify synthetic lethal interactors with *VHL* mutation were reported: Bommi-Reddy et al. performed a shRNA screen targeting 88 different kinases in 786-O and RCC4 cells, and identified three potential candidates (*CDK6*, *MET*, and *MAP2K1*) [38]. And Turcotte et al. performed a small molecule screen in *VHL*-deficient SN12C RCC cells and identified a compound that induces autophagy [39]. We were able to validate our compound in a clinically-relevant tumorgraft model of ccRCC, which is an important addition to prior screens. The identification of HHT, an already FDA-approved drug, allows for relatively fast and more cost-effective “repurposing” of the drug to treat a different cancer type: ccRCC.

## MATERIALS AND METHODS

### Cell lines, transfection, and transduction

786-O cells were purchased from ATCC. They are a human ccRCC cell line and lack a functional *VHL* gene [40]. The pcDNA-HA empty vector control plasmid (EV; plasmid database p332) and the pcDNA-HA-*VHL* plasmid (*VHL*; p333) were transfected into 786-O cells with Lipofectamine reagent following manufacturer's instructions. The H2B-GFP (p585) and H2B-mCherry (p584) retroviral constructs were transduced as previously described [41].

### Chemical library and screening

We screened roughly 12,800 small molecule compounds, available through the UT Southwestern High-Throughput Screening Core (http://www.utsouthwestern.edu/education/medical-school/departments/simmons/shared-resources/high-throughput/lab.html), including the Prestwick library of all FDA-approved drugs and the NIH Collection of experimental drugs. Sixteen-hundred cells of a 1:1 mixture of 786-O-EV;H2B-GFP (EV^GFP^) cells and 786-O-VHL;H2B-mCherry (*VHL*^mCh^) cells were plated using a Multidrop liquid dispenser (Thermo-Fisher Scientific, Inc.). Twenty-four hours after the cells were plated, the compound library was dispensed at a final concentration of 5 μM using a Biomek FX liquid handler. The screen was performed in the presence of a low concentration of sirolimus (2 nM) that equally affected *VHL*-deficient and *VHL*-reconstituted cells, and modestly reduced cell proliferation. 48 hours later, the cells were washed with PBS, stained with Hoechst 33342 (2 ug/mL in PBS), fixed with formalin, and evaluated by the BD Pathway 855 imager (Bectin-Dickinson, Inc.). Controls for each plate included: 16 wells of GFP and mCherry cells plated at (i) 1:1 ratio and (ii) 0.2:1 ratio (with and without sirolimus).

### Tumorgrafts

Tumorgrafts were generated and propagated as described previously, with tumor samples implanted subcutaneously into the flank of the mouse for drug trials [42]. Briefly, 4- to 8-week-old NOD/SCID mice were anesthetized with isoflurane and implanted subcutaneously with 64 mm^3^ tumor fragments (cohorts 2-20). Once tumors reached an average of at least ~250 mm^3^, drug treatment was begun. The mice were treated with vehicle, 0.7 mg/kg homoharringtonine (HHT) via gavage twice daily, or 0.5 mg/kg rapamycin given by IP injection once every 48 hours. Tumor size (volume) was measured twice weekly and was obtained by multiplying tumor length, width, and depth using calipers. To assess toxicity, mouse weight was monitored once weekly.

### Histology

Sections from tumors were stained with hematoxylin and eosin (H&E) as described previously [22].

### Pharmacokinetic analysis

Female NOD/SCID mice (6-7 weeks) were administered homoharringtonine (HHT) by oral gavage. DMSO was used to wet the compound which was subsequently diluted in normal saline (pH 7.2) prior to administration (final DMSO < 5%). Animals were sacrificed in duplicate by inhalation overdose of CO_2_ at varying times post-dose and bled by cardiac puncture using acidified citrate dextrose (ACD) as the anticoagulant. Plasma was isolated after centrifugation at 9,600 x g for 10 minutes at 4°C. Protein was precipitated from 100 μl of plasma with 100 μl of acetonitrile containing 100 ng/ml of n-benzylbenzamide, which was used as an internal standard (IS), and 0.2% formic acid. After extensive vortexing, samples were incubated for 10 minutes at RT and spun twice at 16,100 x g for 5 minutes. The supernatant was evaluated by LC-MS/MS using a Shimadzu (Columbia, MD) Prominence LC coupled to an AB Sciex (Framingham, MA) 3200 Qtrap mass spectrometer. Extraction conditions were optimized prior to PK analysis for efficient and reproducible recovery over a three log range of concentrations. Analytical standards and quality control samples were prepared in a similar fashion by spiking commercial CD-1 mouse plasma (Bioreclamation, Westbury, NY) with known quantities of HHT. Chromatography conditions were as follows: an Agilent (Santa Clara, CA) C18 XDB, 5 micron packing, 50 × 4.6 mm size column was used for reverse phase chromatography. Buffer A consisted of dH_2_O + 0.1% formic acid and Buffer B consisted of methanol + 0.1% formic acid. Gradient conditions utilized were: 0.01-1.0 min 3% B, 1.0-1.5 min gradient to 100% B,1.5-3.0 min 100% B, 3.0-3.1 min gradient to 3% B, 3.1-4.1 min 3% B. HHT was detected as the 546.3 to 298.3 transition, and the IS (n-benzylbenzamide) as the 212.1 to 91.1 transition. Data were analyzed using Analyst software (AB Sciex.) A value 3x above the signal obtained in the blank plasma was designated as the limit of detection (LOD). The limit of quantitation (LOQ) was defined as the lowest concentration on the standard curve at which back calculation yielded a concentration within 20% of the theoretical value and above the LOD signal. The LOQ for HHT was 0.5 ng/ml. Pharmacokinetic properties were evaluated using the noncompartmental analysis tool in WinNonlin (Certara, Corp., St. Louis, MO). Sparse sampling was used for data analysis. Terminal half-life was calculated as the ln(2)/λ_z_ where λz is a first order rate constant associated with the terminal (log-linear) portion of the curve. It is estimated by linear regression of time *vs*. log concentration by the software for three or more of the final nonzero data points. T_max_ (time to maximal drug concentration) and C_max_ (maximal drug concentration) were determined by visual inspection. Area under the concentration time curve (AUC_inf_) from time 0 to infinity was determined by linear trapezoidal analysis. Apparent Volume of Distribution (Vz/F) is based on the terminal phase and is calculated as Dose/λz*AUC_inf_ while Apparent Clearance (CL/F) is calculated as Dose/AUC_inf_. Calculation of Absolute Vz and CL requires knowledge of oral bioavailability (F) which is calculated as AUC_oral_/AUC_iv_ X Dose _iv_/Dose_oral_ but was not determined here because an IV PK was not performed.

### Reagents

Rapamycin (sirolimus) (LC Laboratories) was dissolved in MeOH. Homoharringtonine was purchased from Sequoia Research Products Limited. Hoechst 33342 (Life Technologies) was used at 2 μg/mL in PBS. Antibodies: HA (1:1000; Covance), GLUT1 and HIF-2α (1:1000; Novus Biologicals), Bcl-xL and Caspase 9 (1:1000; Cell Signaling Technology), and α-Tubulin (1:5000; Sigma Aldrich).

## SUPPLEEMENTARY MATERIAL FIGURES


