# Supplementary material for: High-throughput simultaneous screen and counterscreen identifies homoharringtonine as synthetic lethal with von Hippel-Lindau loss in renal cell carcinoma
Source: Oncotarget. 2015 Jul 3;6(19):16951–62. doi: 10.18632/oncotarget.4773 (PMC4627284; doi:10.18632/oncotarget.4773)
Supplement: Supplementary file 1 [file oncotarget-06-16951-s001.pdf]

High-throughput simultaneous screen and counterscreen identifies homoharringtonine as synthetic lethal with von hippel-lindau mutation in renal cell carcinoma

Supplementary Material

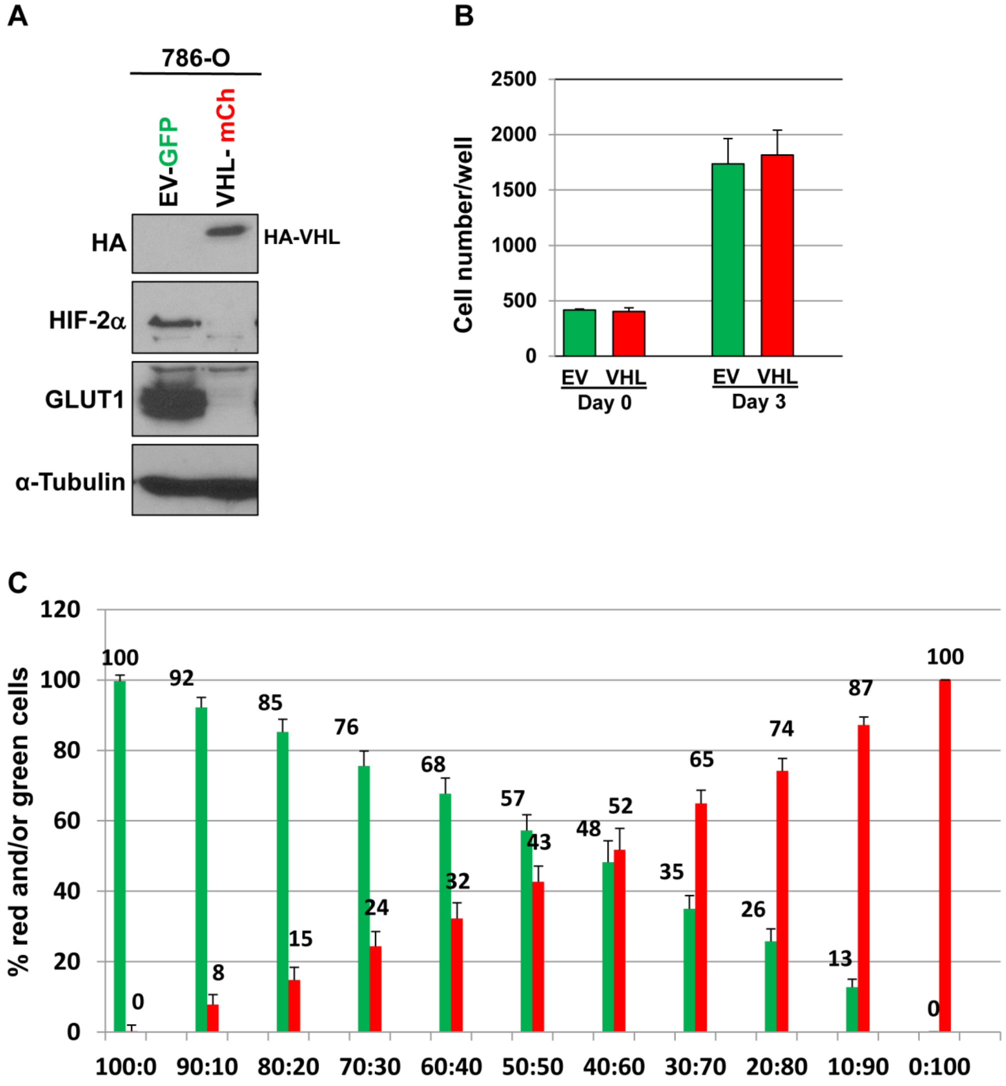

Supplementary Figure 1. VHL reconstitution behaves as expected and does not affect cell proliferation. A. Western blot analysis of HA-tagged VHL (HA-VHL), HIF-2α, and GLUT1 expression in 786-O EV-HA;H2BGFP (EV-GFP) and VHL-HA;H2B-mCherry (VHL-mCh) cells. α-Tubulin was the loading control. **B.** An equal number of EV-HA;H2B-GFP (EV) or VHL-HA;H2B-mCherry (VHL) 786-O cells were plated, and cell numbers counted using the BD Bioimager at Day 0 and Day 3 (n=16 wells for each). **C.** The indicated ratio of cells was plated per well and fluorescence signal measured with the BD Pathway Bioimager (n=16 for each ratio). Error bars are standard deviation.

A

Description	Number of Compounds
NIH Collection	450
Prestwick Library	1,100
Diversity Subset	8,000
UTSW Biochemistry Library	1,010
UTSW Natural Product Collection	2,240 fractions
TOTAL	12,800

B

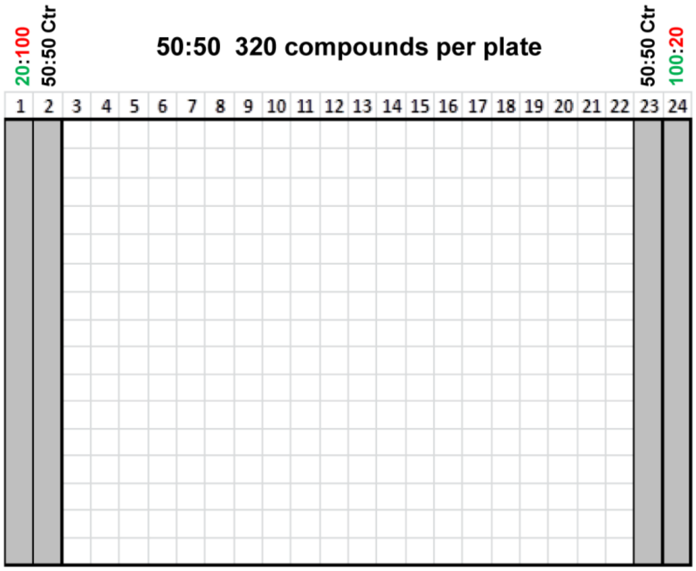

Supplementary Figure 2. Screen library and plating schema. A. The pilot screen was carried out using a small molecule chemical compound library available through UT Southwestern's High Throughput Screening Core. **B.** Plating schema showing layout of controls and samples on the 384-well screening plate.
